# Relationship Between Government Expenditures on Health and Residents' Consumption: New Evidence From China Based on the Bootstrap Rolling-Window Causality Test

**DOI:** 10.3389/fpubh.2021.710147

**Published:** 2021-07-23

**Authors:** Ting-Yu Jiang

**Affiliations:** School of Business, Guilin University of Electronic Technology, Guilin, China

**Keywords:** rolling window, bootstrap, government expenditure on health, consumption, time-varying causality

## Abstract

This paper explores the necessity of expanding government expenditures on health (GEH) from the perspective of promoting residents' consumption (RC). It employs bootstrap full- and subsample rolling-window Granger causality tests to investigate the mutual causal influence between GEH and RC. It finds that GEH have a positive impact on RC in some periods and a negative impact in other periods. The positive effect from GEH to RC reveals that Chinese governments at all levels should continue to increase GEH, narrow the gap between their medical and health investments and those of developed countries', directly reduce current medical expenses of residents, and increase the immediate consumption of residents. However, this opinion cannot always be upheld because a negative impact from GEH to RC also exists. The current paper shows that the government should improve the efficiency of the use of health expenditures; effectively shorten the time lag of government health fiscal policies; and promote the positive effect of government health expenditures on RC.

## Introduction

High saving rate (relative to its GDP) of China used to be a hot issue worldwide and was blamed for causing global trade imbalances. A widely held view is that despite remarkably high level of growth of China, the share of residents' consumption (RC) in the total expenditure has been low and declining due to the high and still-rising saving rate of Chinese households, as uncertainty over the provision of pensions, healthcare, and education costs have increased since the mid-1990s (1). Increasing health expenditures leads to a large amount of precautionary savings. As a result, plenty of income is saved rather than consumed, as individuals need savings to fight against uncertainty, especially that relating to health problems. The lack of domestic consumption is believed to be one of the major reasons for external imbalance problem of China. Government spending can be used to stimulate RC. A shock to government direct expenditures has positive and relatively long-lasting effects on private consumption and output (2). Heppke-Falk et al. (3) analyzed the effects of fiscal policy in Germany on the basis of cash data and found that direct expenditures increase output and private consumption on impact. Devereux, Head, and Lapham (4) studied the impact of government spending on the macroeconomy under the assumption of increasing returns to scale and oligarchic competition and found that an increase in government spending will lead to an increase in the level of total output and that an increase in the level of total output will lead to an increase in the real wages of workers, which leads to the substitution of consumption for leisure; thus, an increase in government expenditures leads to an increase in private consumption. Some empirical studies have identified a complementary relationship between government expenditures and household consumption. The strength of this complementary relationship is negatively related to the size of the government. Increased government expenditures will increase the marginal utility level of household consumption, thereby increasing the level of household consumption expenditures (5). However, the effects from government expenditures to consumption are not always positive. Bailey (6) studied the relationship between government expenditures and private consumption and concluded that there may be a certain substitution relationship between government expenditures and private consumption, i.e., the crowding-out effect. Government expenditures are similar to an input in the private production process (7). They have a positive consumption and output effect. A short-term increase in government expenditures will lead to a temporary increase in output and consumption; however, the increase in output and consumption is less than that of the government. Although, a long-term increase in government expenditures still has a positive output and consumption effect, this output and consumption effect is lower than the output and consumption effect of an increase in government short-term expenditures. There is a certain degree of crowding out of consumption and output (8). Amano and Wirjanto (9) used the relative price method to estimate the intertemporal elasticity of substitution between US government expenditures and household consumption. They believed that an increase of 1 unit of government expenditure in the United States would lead to a decrease of 0.9 units of household consumption. The relationship between government expenditures and consumption can help the government find a way to excavate consumption.

This paper explores the necessity of the government to increase its expenditures on health to stimulate consumption. First, consumption is affected by government expenditures on health (GEH). Insufficient public health expenditures and imperfect public medical systems will increase current medical expenses of residents and reduce their disposable income. At the same time, insufficient expenditures, and imperfect medical systems will also increase risk expectations of residents and thus their precautionary savings to prevent future disease risks (10). After analyzing the panel data of 24 OECD countries from 1990 to 2008, the International Monetary Fund (IMF) reported that government health expenditures comprise an important factor in determining the household savings rate; every increase of one yuan in health expenditures by the government will boost the consumption of urban residents by two yuan (11). For another, GEH may be triggered by the fluctuations in RC. However, the interaction mechanism between GEH and RC may shift with time, which may be quite different from the findings of prior studies. Previous studies have seldom focused on the time-varying factor of parameters, but this problem is worth serious attention.

The current study differs from earlier literature in the following aspects. First, since the reform and opening up, a very prominent phenomenon in China has been the low consumption rate and high investment rate (12). The economy in China has long faced bottlenecks in consumption, the imperfection of the social security systems is an important constraint (13), and public health care is an important part of social security. The basic social medical insurance system is mainly funded by the government, with payments from enterprises lagging behind. In contrast to maximizing their own objectives, hospitals do not have strong incentives to provide quality care at the lowest cost. As a result, the price of medical care is higher than it needs to be, which places a heavy burden on individuals (14). Insufficient government spending on public health may be one of the factors that has contributed to the low consumption rate of Chinese residents in recent years. Thus, analyzing the relationship between public health expenditures and consumption can not only present a new perspective for explaining low consumption rate of China but also provide a reference for the government to optimize the layout of medical and health expenditure structures and deepen medical and health reform. Second, this study contributes the use of bootstrap subsample rolling-window causality test to reveal the time-varying causality between GEH and consumption, while previous studies have merely applied the full-sample causality test. It is assumed that single causality is preserved in all periods, which is the limitation of the full-sample causality test. The current study can provide suggestions for the government to invest in the field of public health at a suitable time using the full- and subsample rolling-window test.

This paper proceeds as follows. Section Literature Review describes the literature review. Section Government Expenditure and RC discusses the theoretical model of government expenditure and RC. Section Methodology describes the methodology, and Section Data contains the data. Section Empirical Results provides the empirical findings. Section Conclusion provides the conclusion.

## Literature Review

Many discussions have emerged in the previous literature regarding the necessity of increasing government social spending to lower the level of precautionary savings and thus increase consumption. Some studies support the opinion that government spending on health has a positive impact on household behavior. Barnett and Brooks (15) found that each additional yuan in GEH could increase urban consumption by 2 yuan. The reason for this is that higher GEH reduces household savings. Therefore, the government should broaden the coverage of public health care. Baldacci and Callegari (16) conducted a survey of Chinese household income and discovered that an increase in government social expenditures can make household consumption in China move in the same direction. The study revealed that a 1% of GDP increase in public expenditures would bring a 1.25% of GDP increase in household consumption, supposing that public expenditure is equally distributed in education, health, and pension. Chou et al. (17) pointed out that by reducing the risk of large out-of-pocket medical expenses, comprehensive social health insurance may reduce household motivation to engage in precautionary behaviors such as saving. However, this view is not always upheld. Yang et al. (18) used the panel data of 27 provinces in China (except Beijing and Shanghai) and found that public expenditures on health have a positive impact on the consumption tendency of rural residents in the eastern region but a negative impact in the central region. Meanwhile, such expenditures were found to have no significant impact on consumption propensity of Western farmers. Wang (19) used data from the Inner Mongolia Autonomous Region and established an empirical model to study the impact of public expenditures on health, education, and social security on the consumption rate of ethnic minorities; the analysis showed a trend of first increasing and then decreasing. Using data for the Spanish economy, Fernández and Hernández de Cos (20) found a positive relationship between government expenditures and output in the short term; however, in the medium and long term, public spending expansionary shocks were found to instead be associated with higher inflation and lower output.

The way in which RC influences GEH can be explained from the perspective of taxes. China has formed a tax revenue structure with a turnover tax as the main body. The turnover tax revenue must be derived from consumption because the bearer of the turnover tax burden is the ultimate consumer. Retail sales of social consumer goods, sales of production materials, and total imports are the “troika” that form tax revenue, but the three make different contributions to tax revenue. In terms of value-added tax alone, the contribution rate of wholesale and retail sales value-added to value-added tax is 10–20%, and the growth rate of value-added tax revenue is faster than the growth rate of sales value-added. If a consumption tax is included, the retail sales of consumer goods will make a greater contribution to tax revenue. The contribution of production sales to the value-added tax has always been ~5%; the contribution of import trade to the value-added tax revenue is ~9%, and the contribution to the consumption tax is ~0.1%. The total of the two is <10%. In other words, wholesale and retail sales contribute the most to tax revenue, followed by import trade volume, and production sales contribute the least. Therefore, from the perspective of increasing tax revenue, it is undoubtedly more effective to stimulate domestic social consumption and increase wholesale and retail sales. Jiang (21) also showed that expanding the total sales of final consumer goods in the market and stimulating total social consumption will make an indispensable contribution to tax revenue. This outcome indicates that tax expenditures comprise an instrument i.e., frequently used when a government wishes to achieve certain economic and social effects (22). Hence, consumption may have an impact on government expenditures.

## Government Expenditure and RC

We use the theoretical model constructed by Liu and Wang (23) to explain the relationship between the government expenditures and RC. Assume that national income *Y* consists of four sectors: residents' consumption *RC*_*t*_, corporate investment *CI*_*t*_, government expenditure *GE*_*t*_, and international trade net export *INX*_0_.

Therefore, national income can be expressed as follows:

(1) $$\begin{array}{l} Y_{t}=RC_{t}+CI_{t}+GE_{t}+INX_{0} \end{array}$$

Residents' consumption *CI*_*t*+1_ in the next period comes from the national income of the previous period *Y*_*t*_. Therefore, *RC*_*t*+1_ can be regarded as a function of the national income of the previous period Y. For simplicity, it is assumed that the two maintain a strict proportional relationship, which is as follows:

(2) $$\begin{array}{l} RC_{t+1}=\omega Y_{t} \end{array}$$

where ω is the proportion of RC of national income in period t, i.e., marginal propensity of residents to consume, and it is subject to ω ϵ (0,1).

Enterprise investment is a function of consumer spending propensity of residents. As a leading variable, assume that enterprise investment in period t and the increase in enterprise consumption (the difference between consumption in period t and period t-1) are in a fixed proportion, as follows:

(3) $$\begin{array}{l} CI_{t}=\mu \left(RC_{t}-RC_{t-1}\right) \end{array}$$

where μ is the fixed ratio between the investment amount of the enterprise and the incremental consumption of the enterprise, and it is subject to (0, +∞).

Government expenditures have played an increasingly important role in increasing or decreasing the national income. Therefore, residents pay more attention to the impact of government expenditures on their own consumption every year, and they anticipate their own consumption in the next period based on government expenditures in the previous period (24). *RC*_*t*+1_ can be expressed as follows:

(4) $$\begin{array}{l} RC_{t+1}=RC_{t}+\beta \left(GE_{t}-GE^{*}\right) \end{array}$$

Where, β is the marginal tendency of government expenditure, and it obeys β ϵ (0,1). GE^*^ is the expected value of government expenditure of residents, and the expected value remains constant for a certain period of time. When the actual value of GE^*^ is greater than the expected value, according to Equation (4), residents will reduce their propensity to consume in the next period; when the actual value of GE^*^ is less than the expected value, according to Equation (4), residents will increase their propensity to consume in the next period. Suppose the net exports of the international trade sector *INX*_0_ are constant.

Combining (1), (2), and (3) and eliminating *Y*_*t*_ and *CI*_*t*_, we obtain:

(5) $$\begin{array}{l} \frac{RC_{t+1}}{\omega }=RC_{t}+\mu \left(RC_{t}-RC_{t-1}\right)+GE_{t}+INX_{0} \end{array}$$

Subtract *RC*_*t*_ from both sides of equation (5), we get:

(6) $$\begin{array}{l} RC_{t+1}-RC_{t}=\left(\omega -1\right)RC_{t}+\mu \;\omega \left(RC_{t}-RC_{t-1}\right)+\omega GE_{t} \end{array}$$

Substituting equation (4) into equation (6) and combining similar terms, we obtain:

(7) $$\begin{array}{r} RC_{t}-\frac{\omega \mu }{\omega \mu +\omega -1}RC_{t-1}+\frac{\omega -\beta }{\omega \mu +\mu -1}GE_{t}\\ +\frac{\beta }{\omega \mu +\omega -1}GE^{*}+\frac{\omega }{\omega \mu +\omega -1}INX_{0}=0 \end{array}$$

Equation (7) is a first-order difference equation about *RC*_*t*_, and its cosine solution and special solution are as follows:

(8) $$\begin{array}{r} RC_{t}=M{\left(\frac{\omega \mu }{\omega \mu +\omega -1}\right)}^{t}+\frac{\beta -\omega }{\omega -1}GE_{t}-\frac{\beta }{\omega -1}GE^{*}\\ -\frac{\omega }{\omega -1}INX_{0} \end{array}$$

Where, $M=RC_{0}-\frac{\beta -\omega }{\omega -1}GE_{0}+\frac{\beta }{\omega -1}GE^{*}+\frac{\omega }{\omega -1}INX_{0}$.

From equation (9), it can be seen that the equation on the left is the consumption of the consumers in period t, and the equation on the right is divided into two parts. The first half of the equation on the right, $M{\left(\frac{\omega \mu }{\omega \mu +\omega -1}\right)}^{t}$, represents the degree of deviation of RC from the equilibrium position on the time path. The second half of the equation on the right, $\frac{\beta -\omega }{\omega -1}GE_{t}-\frac{\beta }{\omega -1}GE^{*}-\frac{\omega }{\omega -1}INX_{0}$, represents the intertemporal equilibrium level of RC, and the last two items are constant.

It can be seen from $\frac{\omega \mu }{\omega \mu +\omega -1}$ that because ω − 1 < 0, $\frac{\omega \mu }{\omega \mu +\omega -1}\in \left(0,1\right)$. Therefore, when *t* → ∞, $M{\left(\frac{\omega \mu }{\omega \mu +\omega -1}\right)}^{t}\to 0$. Its economic significance is that residents will smoothly converge to $\frac{\beta -\omega }{\omega -1}GE_{t}-\frac{\beta }{\omega -1}GE^{*}-\frac{\omega }{\omega -1}INX_{0}$ in an infinite period. Therefore, when *t* → ∞, *RC*_*t*_ can be expressed as follows:

(9) $$\begin{array}{l} RC_{t}=\frac{\beta -\omega }{\omega -1}GE_{t}-\frac{\beta }{\omega -1}GE^{*}-\frac{\omega }{\omega -1}INX_{0} \end{array}$$

Therefore, government expenditure has a stable and continuous positive effect on RC.

## Methodology

### Bootstrap Full-Sample Causality Test

In the case of the violation of stationarity of the standard causality, an asymptotic distribution does not hold. The estimation of the VAR model is difficult in the absence of a standard asymptotic distribution (25, 26). This problem can be solved by following Shukur and Mantalos (27), who indicated that the residual-based bootstrap (RB) method can be used to improve critical values in power and size. In addition, Shukur and Mantalos (28) also proved that small sample revised likelihood-ratio (LR) tests exhibit relatively better power and size. Hence, this paper resorts to the RB-based modified-LR statistic to examine the causality between GEH and RC in China. For the manifestation of the RB-based modified-LR causality test, we deal with the bivariate VAR (p) process as follows:

(10) $$\begin{array}{r} X_{t}=\lambda_{0}+\lambda_{1}X_{t-1}+\ldots +\lambda_{p}X_{t-p}+\lambda_{t}\\ t=1,\;2,\;\ldots \ldots ,T \end{array}$$

When processing subsamples, the RB-based modified-LR technique manifests well-performing characteristics. This study applies the Schwarz Information Criterion to select the optimal lag p. We separate *X*_*t*_ into (*X*_1*t*_, *X*_2*t*_)' and rewrite equation (11) as follows:

(11) $$\begin{array}{l} \left[\begin{array}{c} GEH_{t}\\ RC_{t} \end{array}\right]=\left[\begin{array}{c} \lambda_{10}\\ \lambda_{20} \end{array}\right]+\left[\begin{array}{c} \lambda_{11}\left(L\right)\;\lambda_{12}\left(L\right)\\ \lambda_{21}\left(L\right)\;\lambda_{22}\left(L\right) \end{array}\right]\left[\begin{array}{c} GEH_{t}\\ RC_{t} \end{array}\right]+\left[\begin{array}{c} \varepsilon_{1t}\\ \varepsilon_{2t} \end{array}\right] \end{array}$$

where $\varepsilon_{t}={\left(\varepsilon_{1t},\varepsilon_{2t}\right)}^{\prime }$ is a white-noise process with zero mean and covariance matrix: $\lambda_{ij}\left(L\right)=\overset{p}{\underset{k=1}{\sum }}\lambda_{ij,k}L^{k},\;i,j=1,\;2$ and L is a lag operator determined as $L^{k}X_{t}=X_{t-k}$.

The null hypothesis is trialed by imposing the limitation λ_12, *k*_ = 0 for k = 1, 2, …, p. Analogously, the opposite null hypothesis that GEH does not cause RC is tested by imposing the restriction, λ_21, *k*_ = 0 for k = 1, 2, …, p. The first null hypothesis will be rejected if RC has an impact on GEH, and *vice versa*.

### Parameter Stability Test

This test indicates that a solitary causality exists between GEH and RC in the full-sample period. This is because of the parameter constancy presumption. However, there may be an error in the estimated outcome made under this presumption. It is possible that structural changes may occur in the time series. The Sup-F, Mean-F, and Exp-F tests exploited by Andrews (29), and Andrews and Ploberger (30) can be used to solve this difficulty. A study by Nyblom (31) found that to assess the parameter constancy of a VAR system, the Lc test can be employed as a tool to estimate the chronology of LR statistics.

### Bootstrap Subsample Rolling-Window Causality Test

The rolling-window bootstrap method is utilized to overcome the above problems. This technique is suitable if there is a time-varying character (32, 33) in the causality between GEH and RC. Meanwhile, non-constancy over non-identical subsamples can be noticed using this method (34). The rolling-window technique divides small samples that are rolled sequentially from the start to the end of the full sample. Since the subsamples are of fixed size, the full sample can be transformed into T-l subsamples if a fixed-size window contains l observations. The RB-based modified-LR causality test is conducted on every subsample. The possible changes in the causality between GEH and RC can be observed by collecting the bootstrap *p*-values and LR statistics rolling through T-l subsamples. The effect from RC to GEH is determined by $N_{b}^{-1}\sum_{k=1}^{p}{\hat{\lambda }}_{12,k}^{*}$. Similarly, the effect from GEH to RC is defined as $N_{b}^{-1}\sum_{k=1}^{p}{\hat{\lambda }}_{21,k}^{*}$, where *N*_*b*_ is the number of bootstrap repetitions. Both ${\hat{\lambda }}_{12,k}^{*}$ and ${\hat{\lambda }}_{21,k}^{*}$ are estimations of equation (12). We use 90-percent confidence intervals and keep out the lower 5th and upper 5th quantiles of ${\hat{\lambda }}_{12,k}^{*}$ and ${\hat{\lambda }}_{21,k}^{*}$, according to Balcilar et al. (35).

## Data

In this study, we use monthly data covering the period from 2009:M1 to 2020:M12 to explore whether GEH should be spent to increase RC; we selected this period, because the new medical reform was published in 2009 and spread rapidly to all of China. The specific contents of the Opinions of the Central Committee of the Communist Party of China and the State Council on Deepening the Reform of the Medical and Health System (Zhongfa [2009] No. 6) are as follows: First, fully understand the importance, urgency and arduousness of deepening the reform of the medical and health system. Second, deepen the guiding ideology, basic principles and overall goals of the medical and health system reform. Third, deepen the overall goal of the medical and health system reform. Establish and improve the basic medical and health system covering urban and rural residents, and provide the people with safe, effective, convenient, and inexpensive medical and health services. Since then, a series of medical reforms have occurred around China. In July 2011, the State Council issued a document entitled “Directions on the Establishment of the General Practitioner System.” This document lists principles for establishing the new system to ensure the quality of general practitioners, with a focus on improving their capabilities in clinical practice, standardizing criteria for their training, and creating strict requirements for their licensure and certification. The next phase of reforms, which was announced in detail in 2012, was intended to further deepen the 2009 reforms by enriching insurance benefits, improving portability, encouraging private sector delivery, reforming county-level hospitals, extending the essential medications system to private primary care providers, and further, strengthening population health initiatives. From 2016 to 2017, nationwide networking and cross-provincial medical settlement work in different places was solidly promoted, which provided a better information platform for the comprehensive promotion of integration. In March 2018, the National People's Congress decided that the State Council should establish a “National Medical Security Administration,” which aims to integrate the medical insurance management functions divided into the Human Resources and Social Security Department, the Health and Family Planning Commission, the Development and Reform Commission, and the Civil Affairs Department to form a “major ministry system” management system. In 2019, a reform of the medical insurance system nationwide aimed to “promote the in-depth integration of urban and rural medical insurance, improve the quality of operation, and enhance the security function.” This article uses monthly medical and health expenditures to measure GEH and the total retail sales of social consumer goods to measure RC. To eliminate potential non-stationary, GEH and RC are transformed by taking their natural logarithms and first differences. All the data were collected from the National Bureau of Statistics of China (NBSC).

Residents' consumption changes in the same orientation as that of GEH during the majority of the period. The government announced to the public that increasing fiscal subsidies would be used to meet the basic medical demand starting with the medical reform in 2009; this means that an investment of 850 million was released by the government in year 1 or 2 to deepen the reform. Medical pilot experiments were carried out in hospitals, and access to public medical services was made more equal. The GEH rose as a result of this increase.

On the other hand, RC did not rise in all periods with GEH. GEH declined during 2015, but RC gently rose. The main reason behind this outcome was the outbreak of the global economic crisis in 2015. Therefore, the interlinkage of GEH and RC may vary over time. Table 1 presents the descriptive statistics for GEH and RC. The skewness statistics are all positive. The relevant kurtosis statistics show that the series of GEH has fat tails. In addition, the JB tests of GEH and RC reject the null hypothesis of normal distribution. Hence, it would be misleading to use the traditional Granger causality test. Meanwhile, the full-sample test will lose effectiveness when estimating parameters if the variable has a non-normal distribution. Therefore, we use the bootstrap rolling-window technique to examine the subsamples to solve these problems.

**Table 1:** Descriptive statistics for GEH and RC.

	**GEH**	**RC**
Observations	144	144
Mean	675.357	18901.45
Median	569.215	17983.15
Maximum	2541.200	35893.5
Minimum	58.420	6672.5
Standard deviation	504.579	8161.891
Skewness	1.284	0.255
Kurtosis	4.689	1.913
Jarque-Bera	56.701^***^	8.650^**^

** *Donates significance at the 5% level*.

*** *Denotes significance at the 1% level*.

*Source: Authors' Calculations*.

## Empirical Results

### Bootstrap Full-Sample Causality Test

In this study, we use the VAR model, which is based on equation (12), to examine the causality of GEH and RC. The optimal lag order obtained from the Schwarz Information Criteria (SIC) is 2. We conduct the full-sample tests, and the corresponding results are reported in Table 2. The test results show that GEH does not Granger cause RC according to the bootstrap *p*-values, which is inconsistent with previous research results (2, 14) and the theoretical model in Section Government Expenditure and RC.

**Table 2:** Full-sample Granger causality tests.

**Tests**	**H**_****0****_**: GEH does not Granger cause RC**		**H**_****0****_**: RC does not Granger cause GEH**	
	**Statistics**	***p*-value**	**Statistics**	***p*-value**
Bootstrap LR test	0.439	0.791	15.229^***^	0.000

*** *Donates significance at the 5% level*.

*We calculate the p-value using 10,000 bootstrap repetitions*.

*Source: Authors' Calculations*.

### Parameter Stability Test

A single causal relationship across the entire sample period is considered due to the fixed values of parameters. However, the assumption of parameter constancy does not hold in the presence of structural changes. The results are no longer valid, and the causal link becomes unstable (36). Parameter instability is a major problem (37), and the causality between GEH and RC may vary over time. This study introduced the Sup-F, Ave-F, and Exp-F tests to puzzle out the difficulty. The Lc (38, 39) test can be used to test for all parameters in the overall VAR system. The corresponding results are represented in Table 3.

**Table 3:** The results of parameter stability test.

**Tests**	**GEH**		**RC**		**VAR system**	
	**Statistics**	***p*-value**	**Statistics**	***p*-value**	**Statistics**	***p*-value**
Sup-F	32.774^***^	0.000	41.138^***^	0.000	54.259^***^	0.000
Ave-F	19.702^***^	0.000	21.251^***^	0.000	28.295^***^	0.000
Exp-F	12.588^***^	0.000	17.756^***^	0.000	23.314^***^	0.000
L_c_					0.6974	0.3712

*We calculate the p-value using 10,000 bootstrap repetitions*.

*** *Denote significance at the 1 and 10% levels, respectively*.

*Source: Authors' Calculations*.

As presented in Table 3, structural changes exist in GEH as well as RC. The Ave-F test indicates that there exists one sharp shift in the VAR system at a significance of 1%. Moreover, the results from the Exp-F test indicate that the parameters may evolve at a significance level of 1% in the VAR system. The Lc test indicates that the overall parameter of the system is constant. During the time series of GEH and RC, the parameters are significantly non-constant according to the Sup-F, Ave-F, and Exp-F statistics. This is not in accordance with the results of the full-sample causality test. Therefore, exerting the RB-based subsample test is necessary.

### Bootstrap Subsample Rolling-Window Causality Test

The *p*-values were calculated in equation (12) using the rolling subsample data. The null hypothesis of RB-based modified-LR causality tests is that GEH does not Granger cause RC, and *vice versa*. It is important to choose an appropriate window size (40, 41). The window size needs to be large to obtain reliable results because the results may be imprecise when the window size is too small. On the other hand, the number of scrolls will be reduced if the window size is too large. In summary, a window width of 24 months is selected (42).

### Results and Discussion

**Figure 1:**
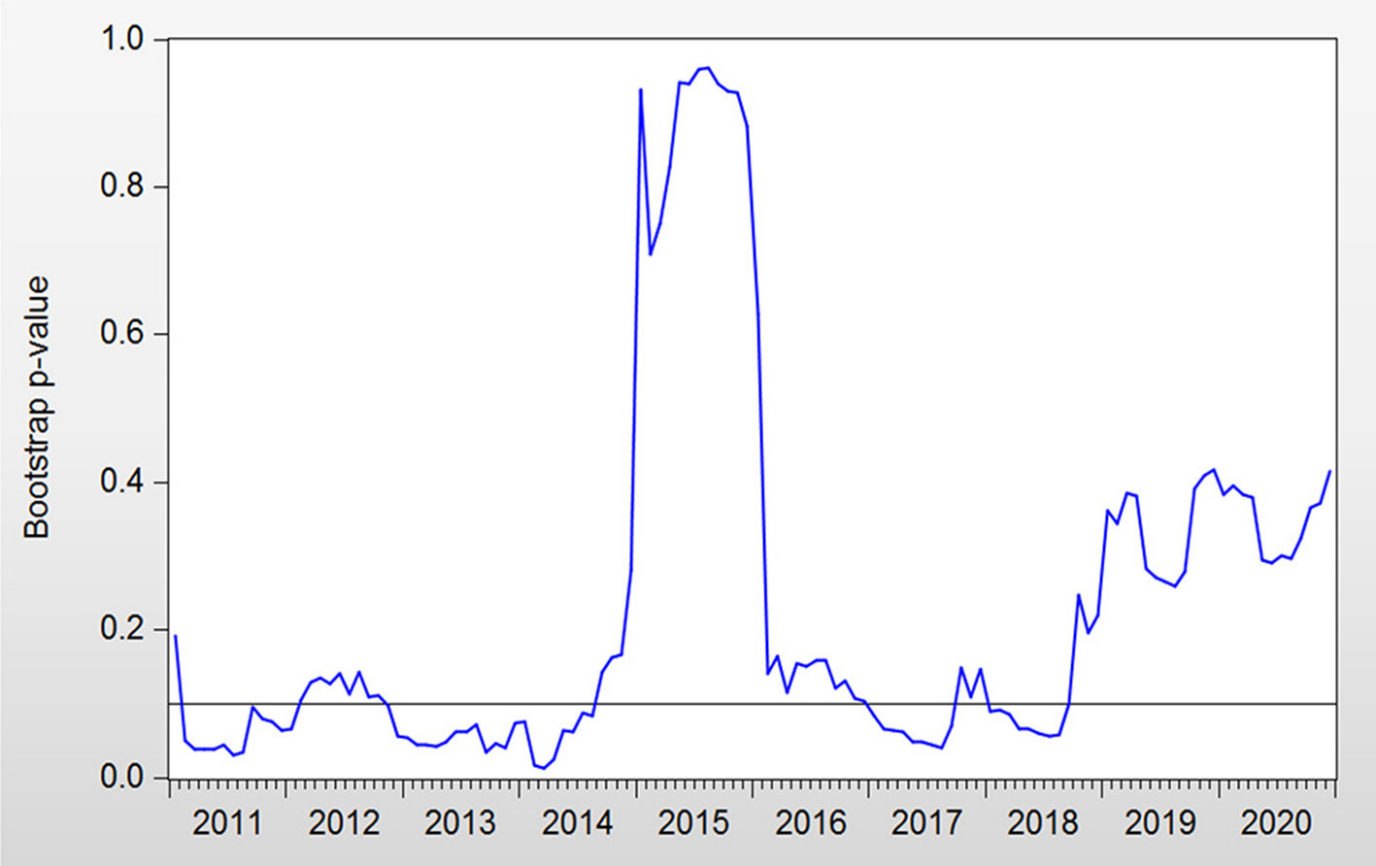
Bootstrap *p*-values of rolling test statistic testing the null hypothesis that GEH does not Granger cause RC.

**Figure 2:**
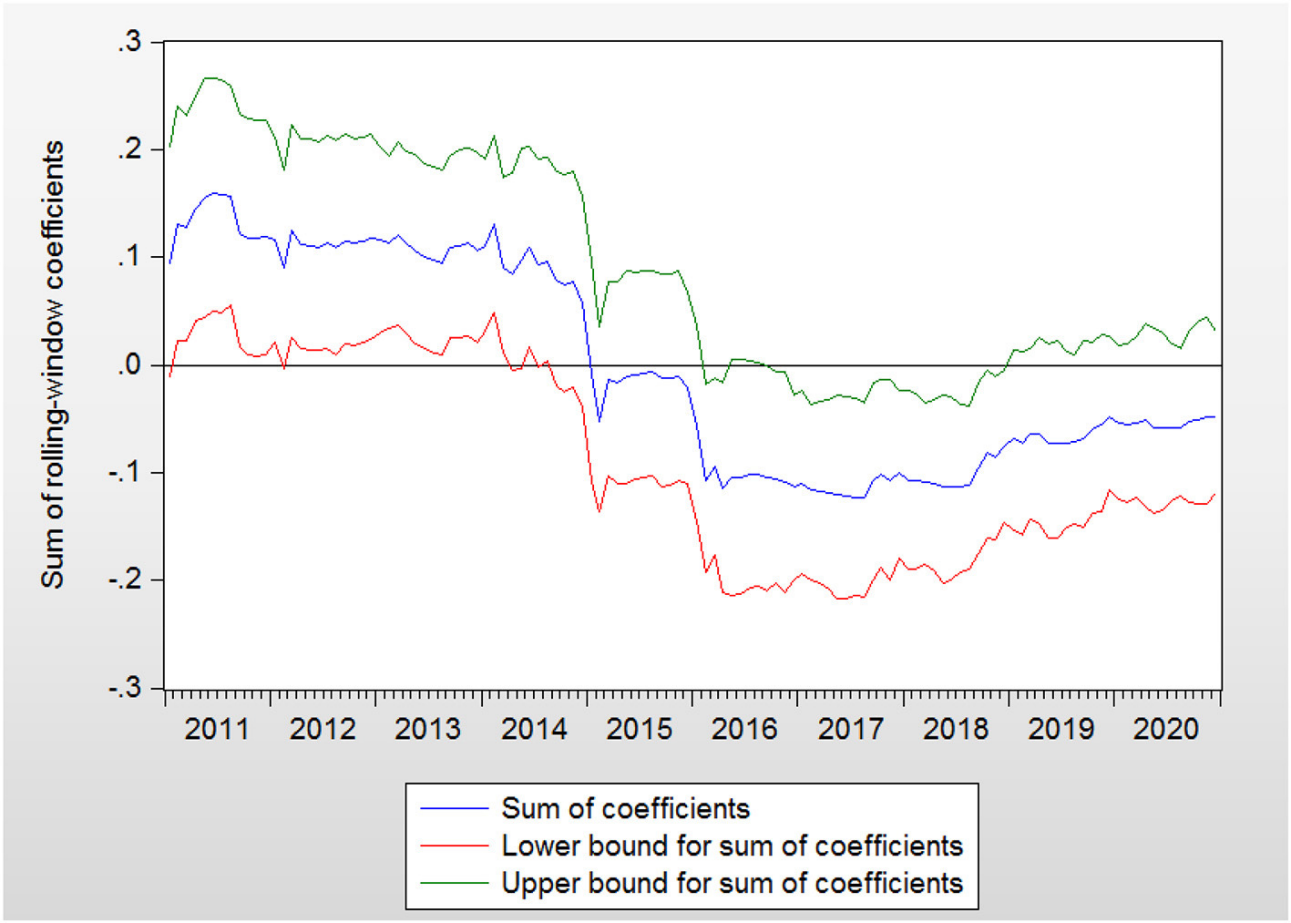
Bootstrap estimates of the sum of the rolling-window coefficients for the impact of GEH on RC.

The bootstrap probability value is presented in Figure 1. GEH affects RC during the periods of 2011:M2–2012:M2, 2012:M6–2014:M8, 2017:M1–2017:M10, and 2018:M1–2018:M9. The orientation of the impact from GEH to RC is presented in Figure 2. The positive effects are in the periods of 2011:M2–2012:M2, and 2012:M6–2014:M8, while the negative effects are in the periods of 2017:M1–2017:M10 and 2018:M1–2018:M9.

There are several periods of positive effects. GEH can be shown to increase RC in these periods. During 2011:M2–2012: M2, there were plenty of medical reforms, including urban community health service organization construction, the completion of the rural three-level health service network, and the improvement of the supply and safety supervision system of essential medicines, which may be the reasons that higher health spending reduced the level of precautionary savings in this period. In turn, a lower cost on health will cause a higher budget for other spending and thus lead to a rise in RC (43). In contrast, when GEH has been reduced, RC has declined as well. The positive association between GEH and RC in 2011:M2–2012:M2 confirms the conclusions of previous studies (44). This is also the case in another period. In 2012:M6–2014:M8, there were many medical reforms around China, such as hierarchical diagnosis and multipoint practice, an increase in resident medical insurance, new rural cooperative medical insurance subsidies, the consolidation of the essential drug system, and the development of critical illness medical insurance. Consequently, we can conclude that GEH can raise consumption. This result supports the theoretical model in Section Government Expenditure and RC. For this reason, GEH are seen to have increased RC during 2011:M2–2012:M2 and 2012:M6–2014:M8.

There are two major reasons for the periods of negative effects. One is the economic sanction (2017:M1–2017:M10), and the other is the outbreak of the Sino–U.S. trade war (2018:M1–2018:M9). In 2017:M1–2017:M10, the GEH continued to grow, but RC was at a relatively low level. This phenomenon can be explained by the economic relationship between United States and China. The U.S.–China trade imbalance was a prime concern of President Trump ever since his election campaign in 2015. The large imbalance has caused considerable pressure on U.S.–China trade relations. The impacts of imports from China on U.S. domestic employment are a major concern (10). The study of Autor et al. (45) indicated that exposure to imports from China exerts a negative influence on the U.S.'s manufacturing employment. As pledged in the presidential election, President Trump aimed to revitalize U.S. manufacturing (46) and retain jobs in the United States by reducing the Sino–U.S. trade imbalance (47).

Therefore, a series of enterprise collapses or redundancies occurred in China. This led to a low level of consumption. Hence, during particular high levels of GEH, a low level of RC can be explained by the influence of the Sino–U.S. economic relationship rather than by GEH. Despite the indicated high level of GEH, the RC still remained low due to the impact of the economic policies that America exerted on China rather than due to GEH. In 2018:M1–2018:M9, the United States and China were locked in a trade confrontation that featured huge tariffs (48). Appreciations in the USD against the target currency and the downside risk (49, 50) of the global economy caused by the trade war (51) were the possible factors driving this phenomenon. Thus, we can see that RC has not risen under the influence of GEH; in contrast, in the case presented herein, we can see that it has fallen. Thus, a negative effect exists from GEH to RC. We can observe that despite the high level of GEH, RC did not increase due to economic reasons. As a result, GEH should not be seen as expanding during the period of 2018:M1–2018:M9. The negative influence from GEH to RC conflicts with the outcome of the theoretical model discussed in Section Government Expenditure and RC.

**Figure 3:**
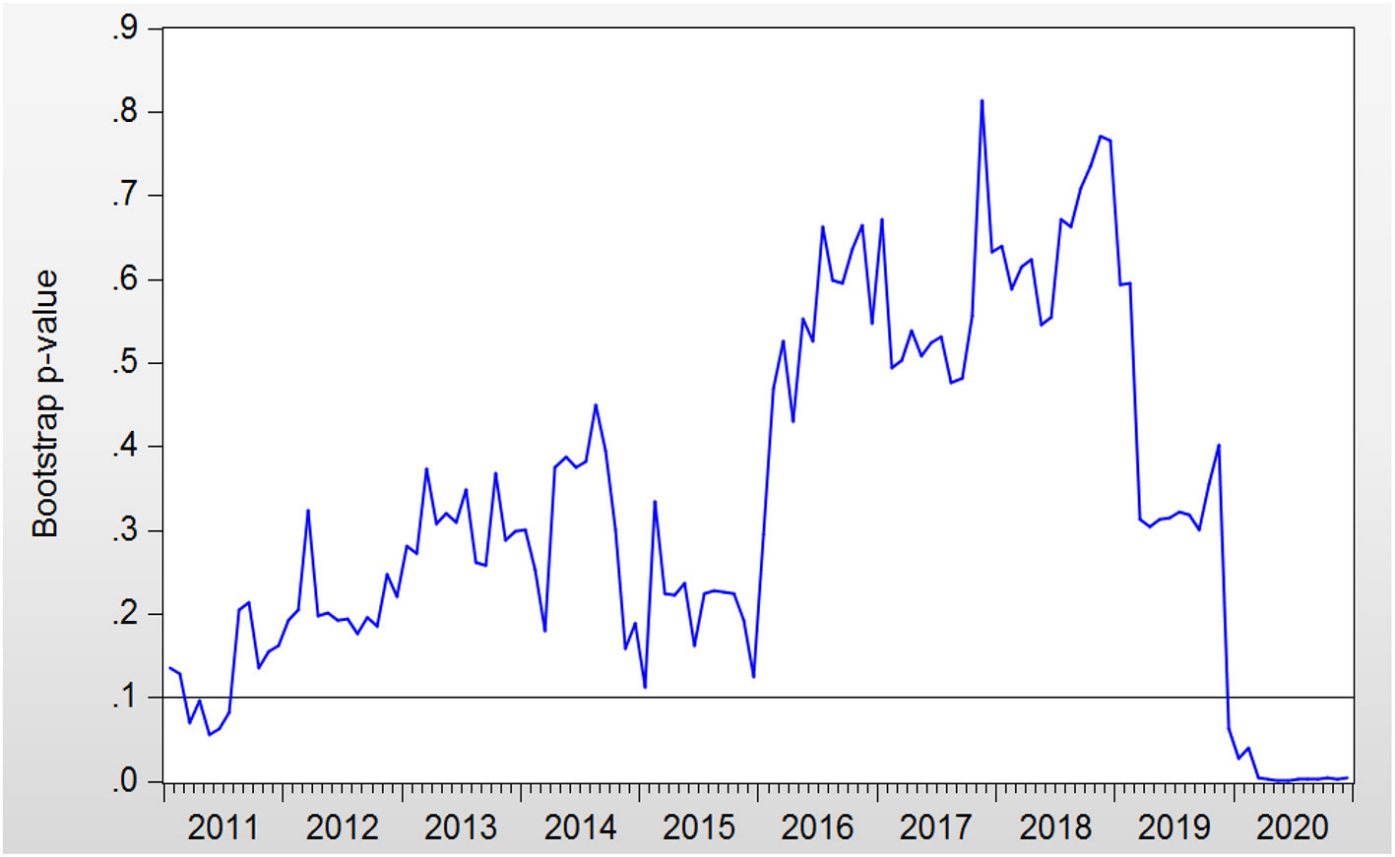
Bootstrap *p*-values of rolling test statistic testing the null hypothesis that RC does not Granger cause GEH.

**Figure 4:**
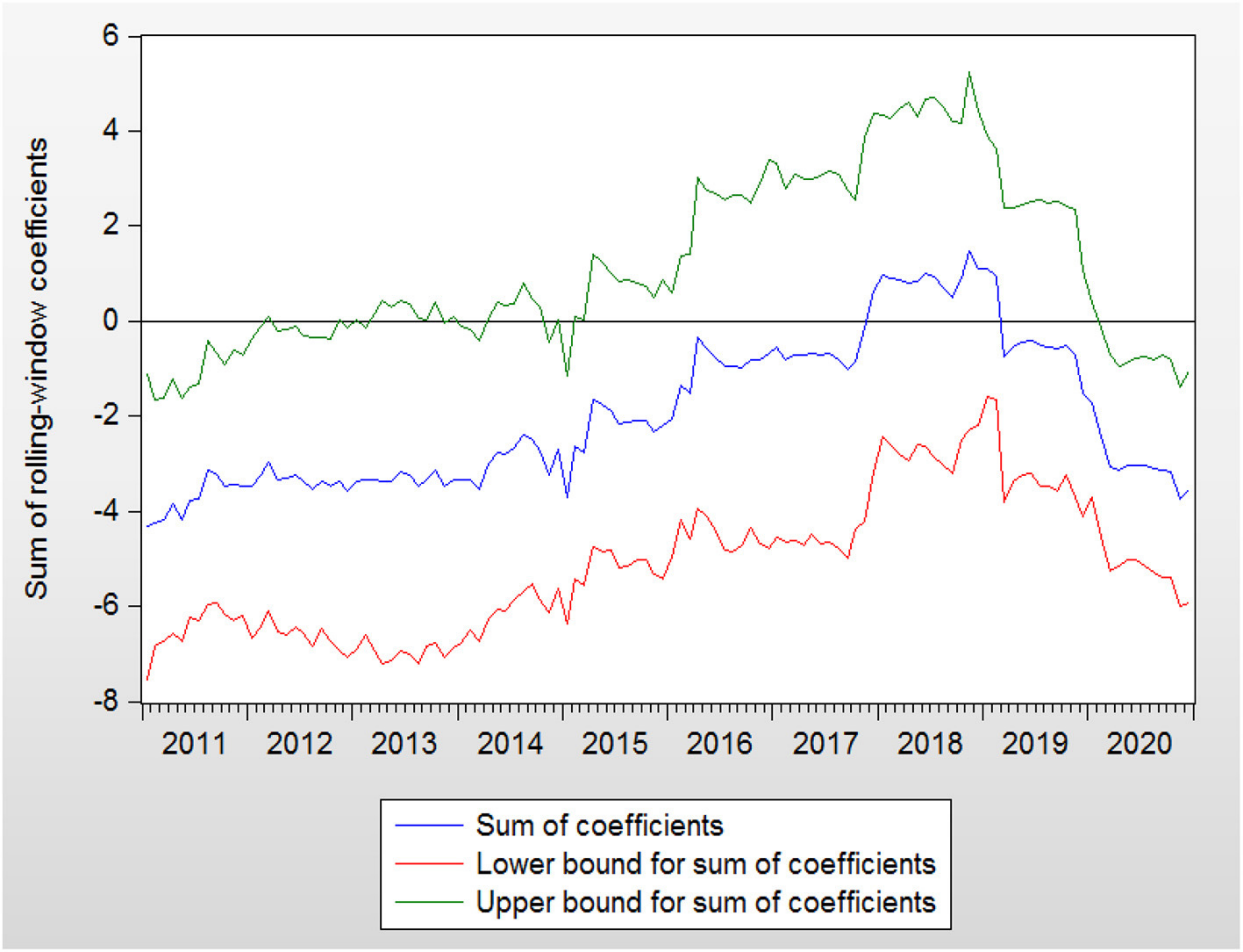
Bootstrap estimates of the sum of the rolling-window coefficients for the impact of RC on GEH.

The bootstrap probability value is presented in Figure 3. RC affects GEH during the periods of 2011:M2–2011:M7 and 2019:M12–2020:M12. The orientation of the impact from RC to GEH is presented in Figure 4.

In the period of 2019:M12–2020:M12, the impact from RC to GEH was negative. GEH rose sharply in this period. In contrast, RC decreased rapidly at the end of 2019 and the beginning of 2020, while GEH continued to rise. Therefore, RC can be seen to cause GEH to move adversely.

In summary, the linkage between GEH and RC is not always consistent. Using the rolling-window causality test method, we confirm that time-varying characteristics exist in these two variables. Meanwhile, the issue of parameter non-constancy is overcome by this method. The matter of whether GEH should be expanded to stimulate RC cannot draw a definite conclusion. RC will move in the same direction as that of GEH in some periods, while this is not the case in some periods. Some factors, such as the economic sanctions from United States and the Sino–U.S. trade war, also play an important role in influencing RC, which does not sustain the theoretical model discussed in Section Government Expenditure and RC. In contrast, if RC subsides, GEH will rise as a result.

## Conclusion

This paper tests the causality between GEH and RC using the rolling-window bootstrap test method. We certify the instability and the structural changes of the causality using the subsample because there is non-causal causality in the full-sample test. Furthermore, we examine the bidirectional relationship and identify the structural changes. There are both significant positive and negative time-varying effects, according to the abovementioned tests. The positive influence demonstrates that a high level of GEH increases RC, because Chinese people like to save in a precautionary manner. Therefore, it is necessary to increase GEH to reduce pressure of residents regarding medical expenses. Nevertheless, RC is affected by not only GEH but also some other economic factors. These recorded results cannot sustain the theoretical model provided in Section Government Expenditure and RC, which claims that RC is positively affected by GEH. Conversely, the negative impact from RC to GEH may indicate that sluggish consumption is the reason for the government to expand its spending. Generally, GEH does have influence on RC. However, GEH should not always be increased in order to stimulate consumption because economic factors such as the national economic situation also have an impact on RC. Therefore, in determining whether to expand GEH to stimulate domestic demand, further, considerations need to be taken into account. Exploring the necessity of increasing GEH and more detailed correlations between GEH and RC could generate recommendations for governments. China is at a critical stage of deepening medical reform; thus, studying the relationship between GEH and RC can be more conducive to promoting the new medical reform from the perspective of social and economic development. Governments can effectively use public expenditures to reduce precautionary savings and thus increase domestic consumption.

## Data Availability Statement

The original contributions presented in the study are included in the article/Supplementary Material, further inquiries can be directed to the corresponding author/s.

## Author Contributions

T-YJ: conceptualization, methodology, software, writing—original draft preparation, visualization, investigation, writing and editing, construct paper structure, and reviewing and editing.

## Conflict of Interest

The author declares that the research was conducted in the absence of any commercial or financial relationships that could be construed as a potential conflict of interest.

## Publisher's Note

All claims expressed in this article are solely those of the authors and do not necessarily represent those of their affiliated organizations, or those of the publisher, the editors and the reviewers. Any product that may be evaluated in this article, or claim that may be made by its manufacturer, is not guaranteed or endorsed by the publisher.
